# A strong direct link from the layer 3/4 border to layer 6 of cat primary visual cortex

**DOI:** 10.1007/s00429-024-02806-3

**Published:** 2024-05-16

**Authors:** Kevan A. C. Martin, Franziska D. Sägesser

**Affiliations:** Institute of Neuroinformatics, ETHZ/UZH, Zürich, Switzerland

**Keywords:** Cat, Visual system, Striate cortex, Cortical circuit, Interlaminar connectivity

## Abstract

The cat primary visual cortex (V1) is a cortical area for which we have one of the most detailed estimates of the connection ‘weights’ (expressed as number of synapses) between different neural populations in different layers (Binzegger et al in J Neurosci 24:8441–8453, 2004). Nevertheless, the majority of excitatory input sources to layer 6, the deepest layer in a local translaminar excitatory feedforward loop, was not accounted for by the known neuron types used to generate the quantitative Binzegger diagram. We aimed to fill this gap by using a retrograde tracer that would label neural cell bodies in and outside V1 that directly connect to layer 6 of V1. We found that more than 80% of labeled neurons projecting to layer 6 were within V1 itself. Our data indicate that a substantial fraction of the missing input is provided by a previously unidentified population of layer 3/4 border neurons, laterally distributed and connecting more strongly to layer 6 than the typical superficial layer pyramidal neurons considered by Binzegger et al. (Binzegger et al in J Neurosci 24:8441–8453, 2004). This layer 3/4 to layer 6 connection may be a parallel route to the layer 3 – layer 5 – layer 6 feedforward pathway, be associated with the fast-conducting, movement-related Y pathway and provide convergent input from distant (5–10 degrees) regions of the visual field.

**Supplementary Information:**

The online version contains supplementary material available at 10.1007/s00429-024-02806-3.

## Introduction

The local circuits of the neocortex have long been the subject of investigation from the era of Ramón y Cajal to the present time. Virtually all attempts to draw a circuit diagram, however, have been qualitative and often based on observations from incomplete reconstructions of neurons, whether from Golgi-stained sections of the early anatomists or from serial electron microscopic reconstruction of small blocks of tissue.

Automated methods of dense reconstruction by serial electron microscopy (SEM) hold out the promise of the most complete wiring diagrams, but despite impressive progress, we still lack a provisional circuit diagram even for mouse primary visual cortex (V1), let alone a complete ‘connectome’ – the ‘Mind of a Mouse’ (Abbott et al. 2020). In the case of bigger-brained higher mammals like the cat or monkey, the sheer dimensions of the circuits are an even greater barrier to dense SEM reconstructions for a single area; individual neurons have local axons that can spread laterally for many millimeters (Gilbert and Wiesel 1979; Martin and Whitteridge 1984).

Functional methods using e.g. glutamate uncaging (Callaway and Katz 1993), paired intracellular recording (Mercer et al. 2005) or optogenetic techniques (Petreanu et al. 2009), are typically carried out in thin slices of cortex, which at best give only a partial picture, because the dendrites and axons are truncated. The lateral connectivity, which is such a prominent feature of local circuits in higher mammals like cat and monkey neocortex, is necessarily incomplete. While these functional approaches allow to study specific cell types with sublaminar precision, summary circuits typically show the vertical flow of information between cortical layers, rather than the details of connectivity at synaptic resolution (e.g. Gilbert and Wiesel 1983b; Douglas et al. 1989; Callaway 1998; Hirsch et al. 1998; Lübke and Feldmeyer 2007*)*.

This leaves sparse reconstructions of individual neurons labeled in vivo presently as the most effective strategy for visualizing the local circuit (da Costa and Martin 2013). Indeed, this approach produced the rare cases of local cortical circuits that are comprehensive and quantitative, such as Binzegger et al.‘s (2004) circuits for cat primary visual cortex (V1, or area 17) and Narayanan et al.‘s (2015) analyses of rat vibrissal cortex. For both cat and rat, the base data were neurons labeled in vivo and reconstructed in order to visualize the full extent of local axonal and dendritic arbors. Decades of correlated light and electron microscopic (LM/EM) studies of mouse, cat, and monkey neurons, has convinced us that counting axonal boutons at light microscope level is an excellent proxy for quantifying the synapses seen using EM. Most boutons form one synapse only, but in some instances, such as the magno- and parvocellular thalamic axons, each bouton makes on average more than one synapse (Freund et al. 1985, 1989). These observations, together with counts of neurons in the different layers, were incorporated to estimate the synaptic ‘weight’ contributed by a given neuron type to the different layers (Binzegger et al. 2004).

The connection between any two neurons was assumed to follow the rule that an axon connects to a target in proportion to the amount of target dendrite and soma present in the territory of the axonal arbor. This rule, pioneered so effectively by Ramón y Cajal, is now known as Peters’ rule (Braitenberg and Schüz 1998). Exceptions to Peters’ rule, i.e. ‘White’s exceptions’, were included (Binzegger et al. 2004). To gauge how completely the reconstructed neurons represented the local circuit, Binzegger et al. (2004) compared the total number of synapses from these 3D reconstructions with an independent count by Beaulieu and Colonnier (1985*)* of the total asymmetric (excitatory) and symmetric (inhibitory) synapses in each main layer. While the match was good for asymmetric synapses in layers 2 to 5, the reconstructed spiny neurons could only account for 10% of asymmetric synapses in layer 1 and 30% in layer 6. Since reconstructions of spiny layer 1 cells were lacking, some mismatch was to be expected, but the fact that 70% of the asymmetric synapses in layer 6 could not be assigned left us with the puzzle of where these inputs to layer 6 originated.

The present study was conducted to discover the sources of layer 6 input by retrograde tracing. The distribution of retrogradely-labeled neurons among different brain areas and cortical layers revealed that layer 6 received its main input from neurons in the local circuit (within area 17) with only minor contributions from thalamus, higher-order visual cortices, claustrum and callosal projections from the homotopic region in the contralateral visual cortex. Unexpectedly, the most prominent label was in neurons at the border between layer 3 and 4 in area 17, which were not previously known for sending a strong projection to layer 6.

## Methods

### Animal preparation

All experiments were performed in agreement with the Veterinary Office of Zurich guidelines under a license granted to K.A.C. Martin and in accordance with the Basel Declaration principles. Data from 10 adult cats (*Felis catus*, weight 3.0 to 5.2 kg, age 9 months to 7 years, 8.5 months, of either sex) were used for this study.

General anaesthesia was induced with a subcutaneous or intramuscular injection of xylazine (~ 0.5 mg/kg, Rompun 2%, Bayer) and ketamine (~ 15 mg/kg, Narketan 10, Vétoquinol or Ketasol 100, Graeub) and first maintained with 1–2% halothane (Arovet) in oxygen/nitrous oxide (50%/50%) and later with intravenous injections of alphaxalone/alfadolone (Saffan, Schering-Plough Animal Health, 50% in Ringer solution, Braun) through a cannula placed in the femoral vein. Sterile surgical techniques were used throughout. The head of the cat was fixed in the stereotaxic apparatus with earbars and a mouthpiece. Lidocaine (Xylocain Gel 2%, Astra Zeneca AG) was applied locally at all pressure points. The eyes were taped close to prevent them from drying out and sometimes additionally moistened with vitamin A lubricant (Bausch & Lomb). The electrocardiogram was continuously monitored and the rectal temperature was kept at 37 °C with a thermistor-controlled heating mat (Harvard Apparatus).

After exposing the bone, a cranial window was drilled (using a Osada micromotor) on the left hemisphere at coordinates 0 to 6 mm posterior to the inter-aural plane (IAP) and 0 to 3 mm lateral to the midline. Horseradish peroxidase (Roche) was freshly dissolved in 2% dimethylsulfoxide (DMSO, Sigma). Fine cuts were made into the dura mater and sometimes the arachnoid to visualize the placement of the injection micro-pipette on the brain surface. Four to five injections of HRP were made unilaterally inside the primary visual cortex (area 17) along the top of the lateral and postlateral gyrus, less than 5° lateral of the representation of the vertical meridian (Tusa et al. 1978). The target depth for layer 6 injections was 1800–2100 μm at an angle of 10° off the vertical (tilted so that the pipette tip pointed medially). Injections were made using glass micro-pipettes with an outer tip diameter of 16–30 μm, either manually with pressure, while monitoring the movement of the meniscus of the solution in the pipette, or iontophoretically with 2–4 µA for 10–20 min. Four cats (cats 2, 7, 8, 10) received additional injections of biotinylated dextran amine (BDA) in layer 3 at a vertical depth of 600–800 μm below the brain surface and several millimeters anterior of the layer 6 injections. BDA (BDA-MW10000, molecular probes) had been aliquoted in electrophoretic solution (0.2 M KCl, 0.05 M Tris Base, pH 7.9) and kept in the freezer at -20 °C.

After the injections, the hole in the skull was closed again with the removed piece of skull secured with dental cement (Kem-Dent and Heraeus Kulzer). The temporal muscle and skin was stitched over the skull and Spersapolymyxin (OmniVision) was applied before the wound was closed with sterile Ethibond Excel thread (Ethicon).

During recovery from anesthesia, the cat received a dose of buprenorphine (Temgesic, Essex Chemie, 0.005–0.01 mg/kg subcutaneous) and amoxicillin (Clamoxyl, GlaxoSmithKline, 100 mg in 0.4 ml aqua ad iniectabilia, antipyrogenic, subcutaneous). In the hours and days after the experiment, the cat was observed closely and given subsequent doses of buprenorphine when necessary.

### Perfusion and histology

Six to fifteen days after the tracer injections, the cat was prepared for a terminal experiment to examine the effect of layer-specific inactivation on visually driven neural activity in the local cortical circuit. At the end of the recording experiments Saffan was injected i.v. until the EEG was flat and the cat was then perfused transcardially with 0.9% NaCl followed by 2.5 l of fixative (4% paraformaldehyde, 15% picric acid, and 0.3% freshly added glutaraldehyde in 0.1 M phosphate buffer). If BDA had been injected, the perfusion was continued with 10, 20 and 30% sucrose (0.5-1 l each). After the perfusion, the brain was cut into coronal blocks in stereotaxic planes. Each cortical hemisphere was cut into three large blocks. These blocks were then transferred to 0.1 M phosphate buffer or 30% sucrose, respectively, and the pia mater was removed. Brain blocks that had sunk in the 30% sucrose solution underwent a freeze-thaw procedure (i.e. held dry in a plastic beaker which was dipped in liquid nitrogen until the block was completely white and then transferred quickly to 0.1 M phosphate buffer) to increase tissue penetration.

Brain blocks were cut coronally at 80 μm with a Microm HM 650 V vibratome and washed first in 0.1 M phosphate buffer and then in TB pH 8 (Tris buffer, 6.06 g Trizma.HCl and 1.36 g Trizma.Base in 1 l ddH_2_O). Sections with BDA were incubated with the ABC kit solution (Avidin and Biotinylated horseradish peroxidase macromolecular Complex kit, Vector Lab) overnight. To visualize the injected tracer as a black stain, all sections were then incubated in 0.6% (NH_4_)_2_Ni(SO_4_)_2_ · 6 H_2_O (ammonium Ni(II) sulfate hexahydrate) and 0.015% DAB (3,3’-Diaminobenzidine) in TB pH 8 for 20 min. The catalyst H_2_O_2_ was added (to a final concentration of 0.005%) and the reaction stopped after 5–10 min by replacing the solution with 0.1 M phosphate buffer. The sections were mounted on gelatine-coated slides, air-dried and stained with Neutral red or Cresyl violet to distinguish the cortical layers based on cell sizes and densities (see below). Micrographs were taken with a Leica DMRB light microscope.

### Neuron counts

Using the Fiji plug-in TrakEM2 (Cardona et al. 2012), the micrographs of the coronal sections were aligned, the injection site volumes were measured and the retrogradely-labeled cell bodies were marked manually on alternate (for cortex) or every fourth (for subcortical areas) sections. The counted neurons were multiplied by the corresponding section sampling factor to yield the total number of labeled neurons per brain.

Cortical layers were defined based on the cellular composition described by O’Leary (1941*)*. The boundaries between the cortical visual areas 17, 18 and 19 were determined according to the description by Otsuka and Hassler (1962*)* and Garey (1971*)*. The criterion that was most readily detectable in the Nissl-stained sections was the thinning out of layer 4b: layer 4 is less granular in area 18 than in area 17. For other cortical area borders, the figures of coronal sections in Tusa et al. (1979), Tusa and Palmer (1980), Symonds and Rosenquist (1984), and Updyke (1986) were further consulted as a reference.

Thalamic nuclei were named as in Updyke (1977*)* and Graybiel and Berson (1980*)*. The representation of the visual field in the dorsal lateral geniculate nucleus (dLGN) and in area 17 were described by Bishop et al. (1962; see also Whitteridge 1973) and Tusa et al. (1978), respectively. The studies by Thuma (1928*)* and Guillery (1970*)* were also helpful in assessing the layers of the dLGN, as was the tracing study by Bullier et al. (1984) in relating connected parts of dLGN and area 17.

For three cats (cats 8–10), the cell bodies were labeled very faintly and were therefore only counted in three alternate sections around the injection site. These cats were not considered when calculating the fraction of labeled neurons (see section ‘Relative input strengths’ below) for separate brain areas, but were included in the proportion of labeled neurons in different layers of the visual cortical areas.

In cats with more anterior injections of BDA in superficial layers, the additional injections were sufficiently far apart from the one to two injections in layer 6, so that there were coronal sections between the injection sites with very few labeled cell bodies. This separation of labeled neurons clustered around the injection sites allowed us to characterize the local input pattern to layer 6 of area 17 from the different layers in areas 17, 18 and 19. In one case (cat 2), there were two large HRP injections in layer 6 at the area 17/18 border, at the representation of the vertical meridian (Tusa et al. 1978), and two smaller BDA injections in layers 3/4 of area 17, in the medial bank of the lateral gyrus. Here subcortical labeling was considered as the source of input to layer 6 because the labeled neurons in the dLGN were clustered at the medial border of the nucleus, exactly at the representation of the vertical meridian (Whitteridge 1973). The superficial injection sites were more anterior in the cortex and more peripheral in respect to the visual representation and no cell clusters were found in the part of dLGN where these more peripheral parts of the visual field are represented. HRP-filled neurons could be distinguished by their distinct granular appearance compared to the more smoothly-filled BDA-positive neurons. The labeled neurons in subcortical structures also had this granular appearance typical of HRP-positive neurons. Possible HRP-positive neurons in the anterior visual cortices at the anteroposterior level of the BDA injections were not counted in cat 2, although some granular labeling was observed in the posterior lateral suprasylvian areas (medial and lateral part, PMLS and PLLS).

In three cats (cats 2, 4, 6), the injection sites in layer 6 was exactly in the border region between areas 17 and 18. For this reason, the proportion of input from area 18 was higher in these cats than in cats with injection sites further away from the area 17/18 border.

### Distance to injection sites

The injection site centers as well as all retrogradely labeled neurons were marked manually on the aligned coronal sections. The z coordinates of the labeled neurons were defined by the coronal section in which it was found (+ 80 μm for each section). The maximal distance to the closest injection site for each labeled neuron was calculated as a Euclidean distance based on these coordinates. Although the Euclidean distance does not necessarily reflect the distance that the retrograde tracer traveled (i.e. the length of the axon), it gives an additional measure of the physical spread of labeled neurons.

### Relative input strengths

To show the relative input strength in terms of number of input neurons, we used the following measure: The fraction of labeled neurons (FLN (Markov et al. 2011)) was defined as the number of labeled neurons in a given area or layer divided by the total number of labeled neurons. Neurons in the injection site itself – inside the volume of tracer diffusion around the center of injection, clearly visible as a black stained background in the tissue – had been excluded. The proportion of labeled neurons per layer was calculated for all cortical areas. The number of labeled neurons per area and cortical layer were analyzed and graphed with Matlab (Version 2017a, Mathworks). Fiji (including TrakEM2), Adobe Photoshop and Illustrator and GIMP were further used to produce the figures.

### Binzegger diagram analysis

In their quantitative map of the local circuit of cat area 17, Binzegger et al. (2004) used neurons reconstructed in 3D to derive the number of synapses for each cell type in each layer. Layers 2 and 3 together were considered as one layer, layer 2/3. To get the total number of neurons per layer, the number of cells under 1 mm^2^ of cortical surface in the binocular part of the visual cortex (Beaulieu and Colonnier 1983) was multiplied by 399 mm^2^, the total surface area of cat area 17 (Anderson et al. 1988). Combining the total numbers of neurons with the known proportions of inhibitory neurons or subtypes of excitatory neurons (mentioned on their pages 2 and 5), Binzegger et al. (2004) calculated the number of neurons per reconstructed neuron type. To obtain the total number of asymmetric synapses that these reconstructed neurons make in layer 6, the number of neurons per type was multiplied by the number of synapses the respective excitatory neuron type makes in layer 6 (see their supplementary Table 5). The 70% unassigned asymmetric synapses arose in the comparison of total synapses from reconstructed neurons with an independent count of total number of synapses (Beaulieu and Colonnier 1985; Table 4). The sum of ‘RA synapses’ (synapses with round vesicles and asymmetric membrane differentiation) under 1 mm^2^ of cortical surface in the binocular region of sublayers 6 A and 6B multiplied by the total surface area of cat area 17 were defined as 100%.

These estimates and calculations served as baseline for exploring the effect of changes in the local circuit on the percentage of unassigned asymmetric synapses in layer 6.

### Reconstructions of neurons found in in-house database

The methods for the intracellular injections and histological processing of neurons in layers 3 and 4 are described in Anderson et al. (1994).

## Results

### Injection sites

**Fig. 1:**
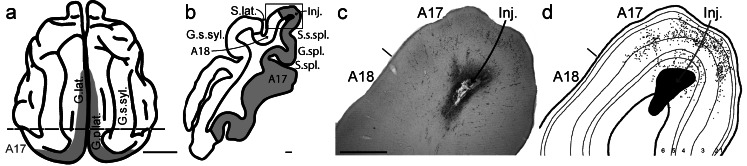
Sample injection site. (**a**) Top-view of cat cortex. The primary visual cortex (V1, area 17, A17) at the back of both hemispheres is marked in gray, running along the lateral and postlateral gyri. G. lat.: lateral gyrus; G. p. lat.: postlateral gyrus; G. s. syl.: suprasylvian gyrus. Image drawn based on Otsuka and Hassler (1962), Abb. 7, Typ I. See also Wilson (1968) for a nice view on the cat gyri and sulci. Scale bar 1 cm (**b**) Coronal section through left hemisphere at the level indicated by the dashed line in (**a**). The injection site (Inj.) in layer 6 of A17 is depicted in black. A17: area 17, primary visual cortex. A18: area 18, secondary visual cortex. S. lat.: lateral sulcus. S. s. spl.: suprasplenial sulcus. G. spl.: splenial gyrus. S. spl.: splenial sulcus. Scale bar: 1 mm (**c**) Close-up of the rectangle in (**b**) with the injection site (Inj.). Center of an HRP (horseradish peroxidase) injection site reacted with Ni-DAB (nickel-diamidinobenzidine) to give a dark stain. Retrogradely labeled neuron somata appear black. Scale bar: 1 mm (**d**) Schematic of the injection site and labeled neuron somata with cortical layers 1 to 6 drawn based on Nissl stainings

To identify the brain areas and cortical layers that provide the unassigned input (Binzegger et al. 2004) to layer 6 of cat primary visual cortex (V1, area 17), we performed targeted injections of the retrograde tracer HRP (horse-radish peroxidase). Successful injections centered in a small patch (0.19 ± 0.18 mm³, min. 0.01 mm³, max. 0.65 mm³) in layer 6 of area 17 at the top of the left lateral or postlateral gyrus, between 1 and 5 mm posterior of the inter-aural plane where layer 6 is thickest (see Fig. 1; Table 1 in Online Resource 1). In each cat, several injections were made along the representation of the vertical midline, starting from just above the representation of the horizontal midline and reaching no lower than 10° below the horizontal midline, based on a reference map (Tusa et al. 1978).

In three cats, the injection sites were slightly more lateral and included area 18. These will be referred to as injections at the area 17/18 border. The most posterior injection was in one of these cats (cat 2). This injection site was located where the posterior part of the suprasylvian gyrus (see Fig. 1a) first appears in the coronal section, where receptive fields just above the horizontal meridian are represented (Tusa et al. 1978). Except for a tiny injection in cat 5 that was slightly lower on the medial bank of the lateral sulcus (well inside area 17), our injection sites in layer 6 were all within 2 mm lateral distance of the area 17/18 border, where the vertical meridian is represented.

**Fig. 2:**
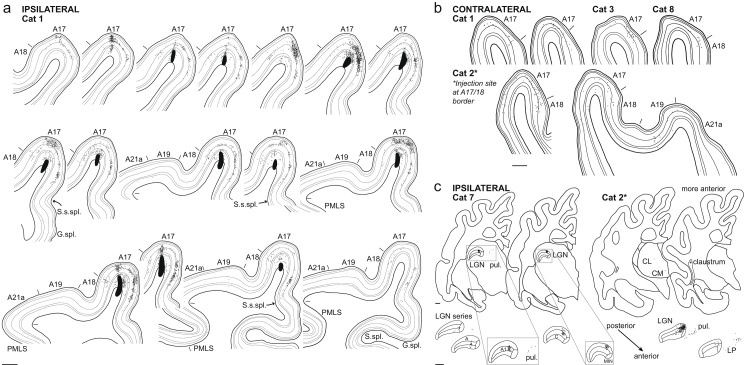
Series of coronal sections. (**a**) Ipsilateral cortex. Series of every fourth coronal section of the visual cortices of one cat (cat 1), from posterior to anterior starting at the top left. Injection sites are shown as large dark blobs and labeled neuron somata as black dots. Fine lines separate cortical layers and short lines above the cortex indicate area borders. Note the spread of neuron clusters in layers 3, 4 and 5 at the suprasplenial sulcus. A17: area 17, primary visual cortex. A18: area 18, secondary visual cortex. A19: area 19. A21a: area 21a. PMLS: posteromedial lateral suprasylvian area. G. spl.: splenial gyrus. S. spl.: splenial sulcus. S. s. spl.: suprasplenial sulcus. Scale bar: 1 mm (**b**) Contralateral cortex. Labeled neurons in coronal sections of the contralateral cortex of four cats. Most callosal neurons were in layer 3 of A17 close to the A17/A18 border. Only in one cat (cat 2) were the labeled neurons in contralateral visual cortices outside A17 and A18. A17: area 17, primary visual cortex. A18: area 18, secondary visual cortex. A19: area 19. PMLS: posteromedial lateral suprasylvian area. A21a: area 21a. Scale bar: 1 mm (**c**) Subcortical nuclei. Labeled neurons in thalamus and claustrum of two cats. The more anterior thalamic nuclei LP and CM only contained labeled neurons in cat 2, which had large injections at the A17/18 border. LGN: lateral geniculate nucleus, primary visual thalamus. A: A lamina of LGN. A1: A1 lamina of LGN. C: C lamina of LGN. MIN: medial interlaminar nucleus of LGN. pul.: pulvinar, secondary visual thalamus. LP: lateral posterior thalamic nucleus. CL: central lateral thalamic nucleus. CM: central medial thalamic nucleus. Scale bars: 1 mm

Labeled neurons were found at multiple cortical and subcortical sites, as illustrated in Fig. 2. Most labeled neurons in cortical areas had the angular somata typical of spiny neurons. Labeled neurons from 28 injection sites in the left hemisphere of ten cats formed the basis for the quantitative results. The total number of labeled neurons ranged from 312 to 29,220 per cat (mean 10,249). Excluding tracer-filled neurons within the injection site itself, the total number of labeled neurons per cat ranged from 224 to 28,600 (mean 9493). The total numbers of labeled neurons are listed in Table 2 (Online Resource 1). An exemplary series of coronal sections including the injection sites of one cat is shown in Fig. 2a.

### Areal distribution of labeled neurons – local input prevailing

We identified all brain areas and cortical layers containing retrogradely-labeled neurons and determined the number of labeled neurons for each of these areas in the stained coronal sections. For each cat we then expressed these numbers in relative terms as the ‘Fraction of Labeled Neurons’ (FLN; Markov et al. 2011), which is the number of labeled neurons in each area relative to the total number of labeled neurons outside the tracer-filled injection sites. The FLN is a measure of the relative input weight from different sources, assuming that all labeled neurons make a similar number of synapses in the target or assuming that the probability of retrogradely labeling a neuron is proportional to the number of synapses it forms at the injection site. The calculated FLN values of different brain areas are shown in Table 3 (Online Resource 1) for each cat.

Labeled neurons were observed predominantly in the ipsilateral hemisphere in several visual cortical areas (areas 17, 18, 19, 21, 20 and PMLS (posterior medial lateral suprasylvian area), see Fig. 2a), in several thalamic nuclei (LGN, pulvinar, central medial, central lateral, lateral posterior, lateral dorsal nuclei) as well as in the claustrum (Fig. 2c). In the contralateral hemisphere, labeled neurons were found in visual cortical areas, mostly in areas 17 and 18 (Fig. 2b).

### More than 80% of labeled neurons lie within area 17 itself

**Fig. 3:**
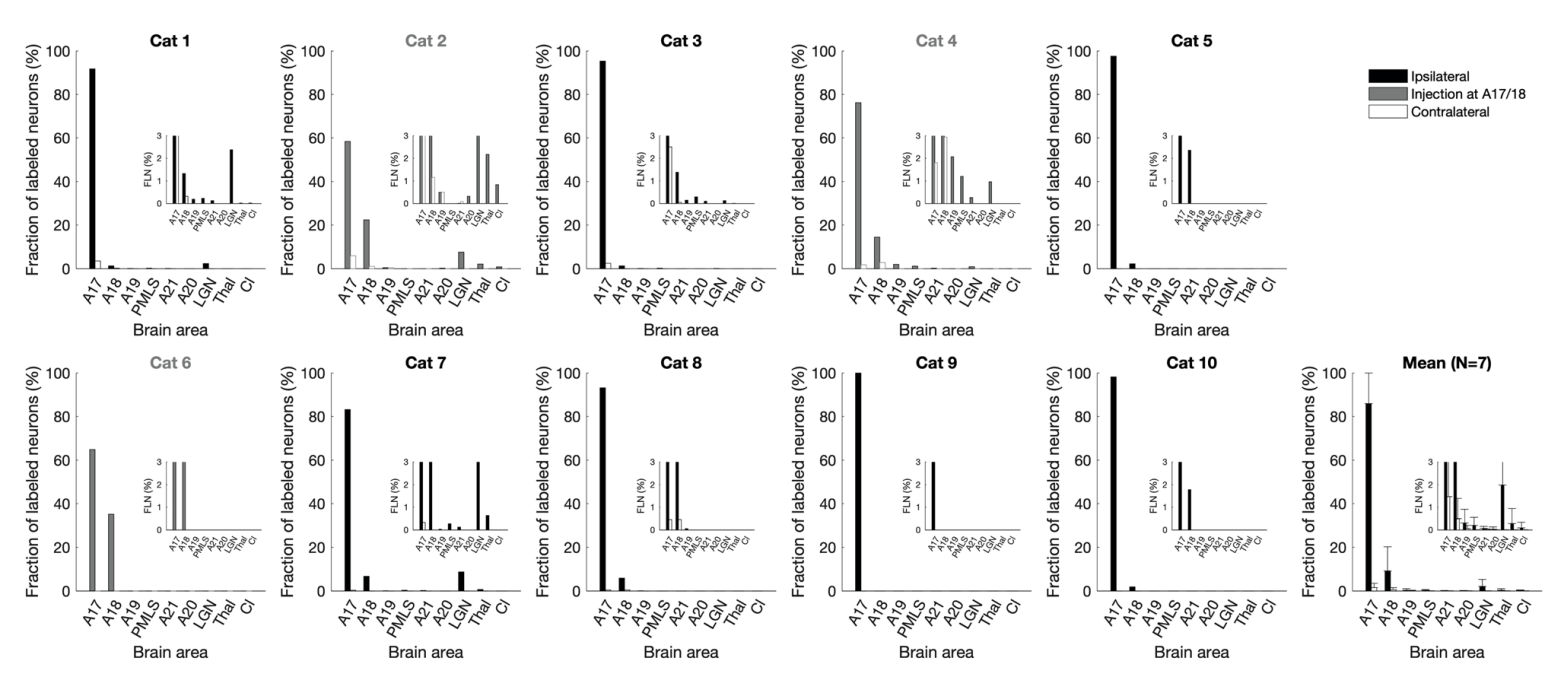
Fraction of labeled neurons (FLN) per brain area. Input weight as percentage of total labeled neurons per brain: Distribution of retrogradely labeled neurons in different brain areas after tracer injections in layer 6 of the left A17. All cats are plotted separately. The last plot shows the average FLN of cats 1–7. Black and gray bars ipsilateral, white bars contralateral to injection site. Gray titles and bars: cat with injections at the A17/18 border. Local input from ipsilateral A17 (and A18) is predominant. Apart from LGN and contralateral A17, all other areas each provide less than 1% of the total input. Insets: Same plot for FLN values below 1%. A17: area 17, primary visual cortex. A18: area 18, secondary visual cortex. A19: area 19. PMLS: posteromedial lateral suprasylvian area. A21: area 21. A20: area 20. LGN: lateral geniculate nucleus, primary visual thalamus. Thal: thalamus, other nuclei. Cl: claustrum. *N* = 7. Error bars: standard deviation

The relative distribution of labeled neurons across these areas is shown in Fig. 3. Clearly the inputs from higher visual cortices, from the thalamus, or the claustrum were not sufficiently numerous to account for the unassigned synapses in layer 6. Instead, the major input to layer 6 came from neurons within area 17 itself with a weight of 81.1 ± 14.1% (Fig. 3, *N* = 7). This local input was even higher if only cats with injection sites clearly inside area 17 were considered: excluding the three cats with injection sites at the area 17/18 border, the FLN of area 17 was 92.0 ± 5.5% (*N* = 4).

### Only sparse input from higher visual cortices

Of the higher-order visual cortices in the ipsilateral hemisphere, area 18 contained by far the largest portion of labeled neurons: the FLN was 12 ± 12% in the neighboring area 18, 0.4 ± 0.7% in area 19, 0.1 ± 0.1% in area 21 (the carnivore analog of primate area V4; Connolly et al. 2012), 0.3 ± 0.4% in PMLS (the carnivore analog of primate middle temporal area MT; Dreher et al. 1996), and 0.04 ± 0.11% in area 20 (Fig. 3, *N* = 7). If only injection sites clearly inside area 17 and not at the area 17/18 border were considered, these input weights dropped to 3 ± 2% for area 18, 0.1 ± 0.1% for area 19, 0.2 ± 0.1% for PMLS, 0.1 ± 0.1% for area 21 and no labeled neurons in area 20 (*N* = 4). Thus, although layer 6 might have been expected to receive substantial input from feedback connections, these higher cortical areas in fact only made a minor contribution to the total input to layer 6.

### Weak thalamic input

The axons from the primary visual thalamus, i.e. the dorsal lateral geniculate nucleus (LGN), arborize densely in layer 4 of the primary visual cortex, and only minor branches of the same axons innervate upper layer 6 (Humphrey et al. 1985; Freund et al. 1985). Our layer 6 injections labeled neurons in the LGN in five cats. The weight of the geniculate projection was 2.8 ± 3.5% (*N* = 7). If the injections in layer 6 were very small (≤ 0.3 mm^3^), no labeled neurons in subcortical areas were found (except cat 7, which had labeled neurons in LGN despite a very small injection site). Of the labeled neurons in LGN, 74 ± 19% were in the A laminae, 22 ± 18% in the C laminae and 3.5 ± 4.0% in the medial interlaminar nucleus (MIN) (*N* = 5). Both large-sized (Y-type) and medium-sized (X-type) cells were labeled in the LGN. The labeled neurons in the C laminae always spread more posteriorly than the labeled neurons in the A laminae (not shown). Most labeled neurons in the C laminae were found in the dorsal-most sub-layer of the C layers (i.e. the magnocellular layer C in the nomenclature of Guillery 1970). These were the large Y-type, not the small W-type cells of the C laminae.

For the cats with injection sites clearly inside area 17, the labeled neurons in the A laminae outnumbered the ones in the C laminae by a factor of 13 (*N* = 3). But for the cats with injection sites at the area 17/18 border, the number of labeled neurons in the C laminae approached the number of labeled neurons in the A laminae (the laminae A to laminae C ratio was 1.4, *N* = 2). In the remaining five cats, no labeled neurons were found in the LGN.

In four cats, there were neurons labeled in thalamic nuclei other than the LGN (see Table 2 in Online Resource 1, ipsilateral thalamus; the different nuclei are not shown separately). A few labeled neurons were found in the pulvinar of three cats and in the central medial nucleus of two cats. In the cat with the largest injection at the area 17/18 border (cat 2), there were additionally a few labeled neurons in the lateral posterior, the lateral dorsal, and the central lateral nuclei and two labeled neurons outside the thalamus: one in the hypothalamus and one in the caudate nucleus (see Fig. 2c). With a total FLN of 0.41 ± 0.76% (*N* = 7), the weight of these extrageniculate thalamic nuclei was minuscule even in comparison with the small number of cells labeled in the LGN.

### Small input from claustrum

Further long-range input came from the dorsal claustrum, where the labeled neurons were clustered around the representation of the central visual field (LeVay and Sherk 1981a). Claustral neurons were only labeled in two cats with big injections (see Fig. 2c). Thus, the claustrum provided a small FLN of 0.1 ± 0.3% (*N* = 7). We did not find any labeled neurons in the contralateral claustrum, although we had injections at the area 17/18 border, which the claustrum innervates in both hemispheres (LeVay and Sherk 1981b).

### Limited callosal input

Apart from the ipsilateral cortex, layer 6 of area 17 received low-weight input from contralateral area 17 (FLN 2.0 ± 2.1%). Labeled neurons in contralateral area 18 provided an FLN of 0.7 ± 1.0% (*N* = 7). Other callosal input was only found in areas 19 and 21 of one cat with an injection at the area 17/18 border and therefore had an accordingly small FLN of 0.07 ± 0.18% and 0.01 ± 0.03%, respectively (see Figs. 2b and 3).

Only the four cats with larger tracer injections (total size > 0.4 mm^3^) had an appreciable percentage of labeled neurons in the contralateral cortex (FLN > 2%). Of these, the two cats with tracer injections at the area 17/18 border had the highest-weight inputs from contralateral cortex with an FLN of 4.8% and 7.7%, respectively (see Table 3 in Online Resource 1).

### Summary of areal distribution of labeled neurons

In summary, 81 ± 14% of labeled neurons were within area 17 itself. Higher visual cortices (mainly the adjacent area 18) provided another 13 ± 12% of the input weight and the remaining few percent came from contralateral visual cortices (3 ± 3%, again predominantly from areas 17 and 18) and subcortical structures (3 ± 4%, mainly from LGN).

This distribution of labeled neurons across brain areas demonstrates that the source of the ‘dark matter’ of the cat primary visual cortex (Binzegger et al. 2004) resides in the local cortical circuit rather than in distant brain areas.

### Laminar distribution of cortical inputs to layer 6

#### Lateral distribution of labeled neurons within area 17

**Fig. 4:**
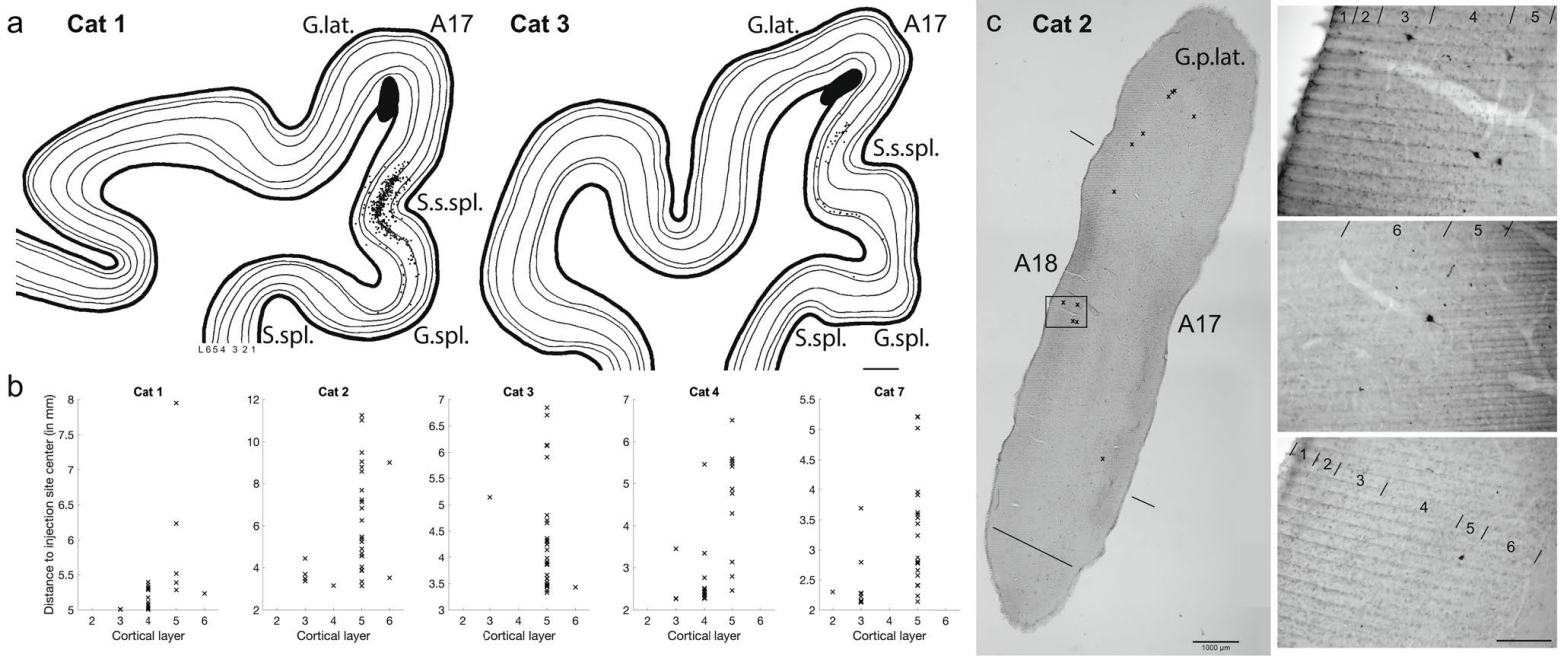
Horizontal spread of labeled neurons in area 17. (**a**) Projection of labeled neurons in area 17 around the suprasplenial sulcus from 18 (cat 1) and 15 (cat 3) coronal sections, respectively. Schematic of the injection site (black blob) and labeled neuron somata (black dots) with cortical layers 1 to 6 drawn based on Nissl stainings. Scale bar: 1 mm. G. lat.: lateral gyrus. G. spl.: splenial gyrus. S. spl.: splenial sulcus. S. s. spl.: suprasplenial sulcus (**b**) The most distant neurons of ipsilateral area 17. For the 5 cats with the largest horizontal spread of labeled neurons, the 30 labeled neurons inside area 17 with the largest Euclidean distance to the closest injection site center are plotted as an ‘x’ in their respective cortical layer. A17: area 17, primary visual cortex. L2: layer 2. L3: layer 3. L4: layer 4. L5: layer 5. L6: layer 6. **(c)** Labeled neurons with large cell bodies in posterior A17 and A18. *Left:* Very posterior coronal sample section of cat 2 with large labeled pyramidal neurons marked with ‘x’. A17: area 17, primary visual cortex. A18: area 18, secondary visual cortex. G. p. lat.: postlateral gyrus. Scale bar: 1 mm. *Right: Top*: Close-up of the neurons in the frame on the left. Numbers denote cortical layers. *Middle & bottom*: Further examples of labeled layer 5 neurons with a large-diameter soma located in A17 within 0.8 mm anterior of the coronal section depicted on the left. Scale bar 200 μm

A striking feature of the data was the extensive horizontal spread of labeled neurons in area 17 (Fig. 4). In four cats (cats 1 to 4), several neurons were labeled at the suprasplenial sulcus. The most laterally displaced spray of labeled neurons even reached the ventral part of the splenial gyrus. Two examples, in which the neurons were projected onto one plane from 18 to 15 coronal sections, respectively, are shown in Fig. 4a.

To identify the layers containing the most distant labeled neurons in area 17, the Euclidean distance of all labeled neurons in area 17 to the closest injection site center was calculated. Although the Euclidean distance is not a measure of the actual axon length of these neurons, the calculated distances provide a means of comparing the shortest physical distance in 3D from the labeled neuron to the injection site center.

In five cats (cats 1, 2, 3, 4 and 7), the largest distances of labeled neurons in area 17 exceeded 5 mm. There, the most distant neuron in area 17 was always in layer 5, but also labeled neurons in the other layers were among the labeled neurons spread horizontally several millimeters distant from the injection site centers (Fig. 4a + b). Due to the small total number of ‘distant labeled neurons’, their relative laminar contribution was not further quantified. According to the visuotopic map in area 17, our injection sites were in all cases at the representation of the vertical meridian or at most 2 degrees azimuth from the vertical meridian. The distant labeled neurons at the suprasplenial sulcus, however, would have receptive fields at least 10 degrees away from the vertical midline (Kalia and Whitteridge 1973; Gilbert 1977; Tusa et al. 1978; Bullier et al. 1984).

The spread of input from more peripheral regions of area 17 to the representation of the vertical midline of the visual field was especially evident in the posterior-most sections of the visual cortex, where the upper visual hemifield is represented (Tusa et al. 1978). In the same five cats, a small number of labeled neurons were found in the most posterior sections in areas 17 and 18 – and even at the ventral borders of these areas (see Fig. 4c). Again these posterior labeled neurons were predominantly pyramidal cells in layer 5. They had large cell body diameters (up to 20 μm, not corrected for shrinkage) and were reminiscent of Meynert cells, although the latter have been reported to have even larger soma diameters of 30–40 μm (Gabbott et al. 1987). An example of a coronal section at the posterior end of visual cortex of cat 2 is given in Fig. 4c. One of these large neurons (marked with ‘x’) was not in layer 5, but in layer 3. The middle and lower image on the right illustrate more examples of large labeled layer 5 neurons in the posterior part of area 17.

Apart from labeled neurons in higher visual areas, the labeled neurons in areas 17 and 18 in the most posterior sections were amongst the ones with the largest Euclidean distance to the injection site centers in the same hemisphere; they were more distant even than the neurons at the suprasplenial sulcus in Fig. 4a. The furthest distance of labeled area 17 neurons ranged from 5.2 mm in cat 7 to 11.2 mm in cat 2 (average of five cats: 7.9 ± 2.5 mm, see Fig. 4b). The most distant neurons in area 18 were also posterior and reached slightly larger distances than area 17 neurons in all cats except cat 3 (average of five cats: 9.5 ± 3.5 mm).

### Ipsilateral visual cortices

**Fig. 5:**
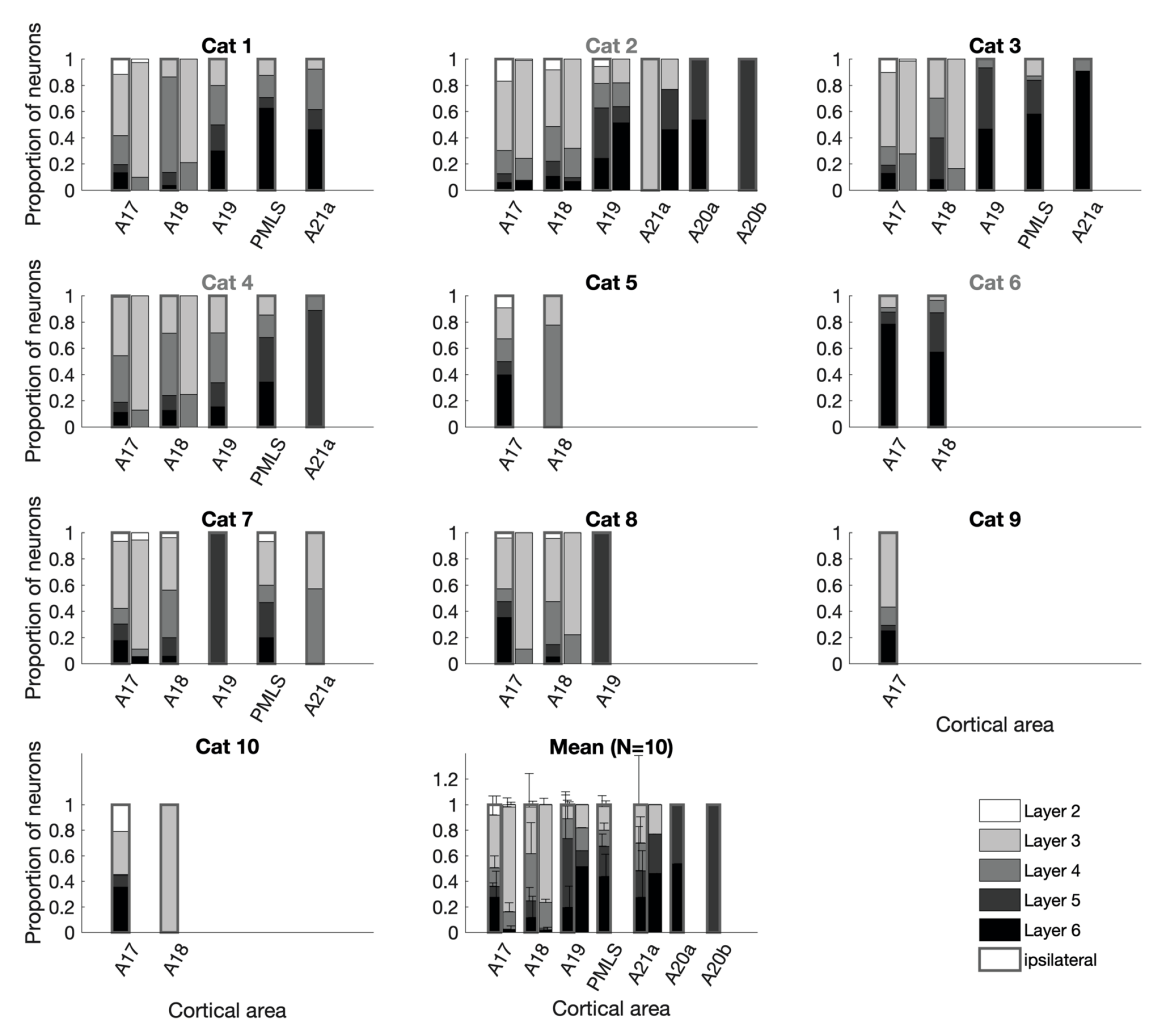
Laminar distribution of neurons in cortical areas. The proportion of labeled neurons in the different layers of each cortical area containing labeled neurons for all cats as well as their average. The framed bars on the left depict cortical areas ipsilateral to and the unframed bars on the right contralateral to the injection site. The areas are sorted from left to right in ascending hierarchical order. Gray title: cats with injections at the A17/18 border. A17: area 17, primary visual cortex. A18: area 18, secondary visual cortex. A19: area 19. A20a: area 20a. A20b: area 20b. A21a: area 21a. PMLS: posteromedial lateral suprasylvian area. *N* = 10. Error bars: standard deviation

Figure 5 shows the distribution of the labeled neurons across the layers of different cortical areas in the ipsilateral and contralateral hemispheres (see also Fig. 2b for coronal sections). Lateral visual areas in the ipsilateral hemisphere were prominent contributors to layer 6. Layer 3 contained the largest fraction of labeled neurons in area 17 (41.3 ± 14.7%), mainly due to many labeled neurons in lower layer 3. Both areas 17 and 18 had more than half of their labeled neurons in layers 3 and 4. In comparison, area 18 had a higher percentage of labeled neurons in layer 4 than area 17 (37.0 ± 24.4% vs. 14.7 ± 9.2%).

In the higher visual areas, in contrast, more neurons were labeled in layers 5 and 6 (73.3 ± 26.0% in area 19, 67.4 ± 13.4% in area PMLS, and 48.3 ± 40.8% in area 21a). The few labeled neurons in areas 20a and 20b were observed exclusively in layers 5 and 6.

### Contralateral visual cortices

As the FLN indicated (Fig. 3), input from the contralateral hemisphere was sparse and was not observed in all cats (six cats had labeled neurons in contralateral area 17, five cats also in area 18 and only one cat had labeled neurons in contralateral areas 19 and 21a). Four of these cats had injection sites on top of the lateral gyrus (clearly inside area 17), two cats at the area 17/18 border. Most of the labeled neurons in the contralateral cortex of these cats were concentrated around the area 17/18 border, which represents the vertical meridian of the visual field. In two cats the labeled neurons were exclusively in layers 3 and 4, but a few layer 2 and layer 6 neurons were observed in the other two cats.

Considering contralateral areas with at least 50 labeled neurons in the samples, the proportion of labeled neurons in the supragranular layers of contralateral area 17 was 81 ± 9% (infragranular proportion: 2 ± 4%) and the proportion of labeled neurons in the supragranular layers of contralateral area 18 was 74 ± 6% (infragranular proportion: 3 ± 5% (see Fig. 5).

Of the two cats that had injection sites at the area 17/18 border, the cat with the smaller injections had contralaterally labeled neurons only in layers 3 and 4 of areas 17 and 18. Labeled neurons spread on both sides of the area 17/18 border more than 2 mm horizontally. The contralateral neurons labeled in the cat with larger injections at the area 17/18 border (cat 2) spread even further (see Fig. 2b). In layers 3, 4 and 6, some labeled neurons in contralateral area 17 were located along the medial bank of the lateral gyrus, at more than 4 mm distance from the injection site at the top of the gyrus. And in area 18, labeled neurons were found more than 2 mm away from the area 17/18 border. A few labeled neurons were also found in layers 2 and 5 on top of the lateral gyrus. In addition, contralateral area 19 contained a few labeled neurons across layers 2 to 6 and area 21a in layers 3, 5 and 6. But in these latter two areas, more than half of the labeled neurons were in the infragranular layers (64% in area 19 (supragranular proportion: 18%) and 77% in area 21a (supragranular proportion: 23%)).

Thus, while most of the projections from the contralateral visual cortex seemed to have a preference for layers 3 and 4 in the vicinity of the area 17/18 border, the labeling pattern in the contralateral visual cortices of cat 2 resembled the labeling pattern seen in the ipsilateral visual cortices.

### Area 17 – Binzegger diagram revised

It seems clear from these new data that Binzegger et al. (2004) underestimated the contribution of local neurons to the total excitatory synaptic complement of layer 6. We thus examined the laminar distribution of labeled neurons and compared it to the laminar distribution of input weights to layer 6 in the Binzegger diagram. In the Binzegger diagram, of the 30% assigned asymmetric synapses in layer 6, nearly 80% originated in the deep layers: 57% came from layer 6 cells and 20% from layer 5 cells. Only 11% came from layer 4 cells and 10% from neurons in layers 2 and 3 (Fig. 8a; for calculation see section ‘Binzegger diagram analysis’ in Methods for calculation).

Compared to these estimated synaptic weights, our new data show a very different laminar input to layer 6 (see Table 4 in Online Resource 1 and Fig. 5, ipsilateral A17 (left bar)). It is not the deep layers, but layer 3 that contains most labeled neurons in area 17, namely 41.3 ± 14.7%, followed by layer 6 with 27.5 ± 20.2%. The third-largest contribution came from layer 4 with 14.6 ± 9.1%. Layers 5 and 2 each contained less than 10% of labeled neurons (8.4 ± 2.6% and 8.1 ± 6.7%, respectively).

**Fig. 6:**
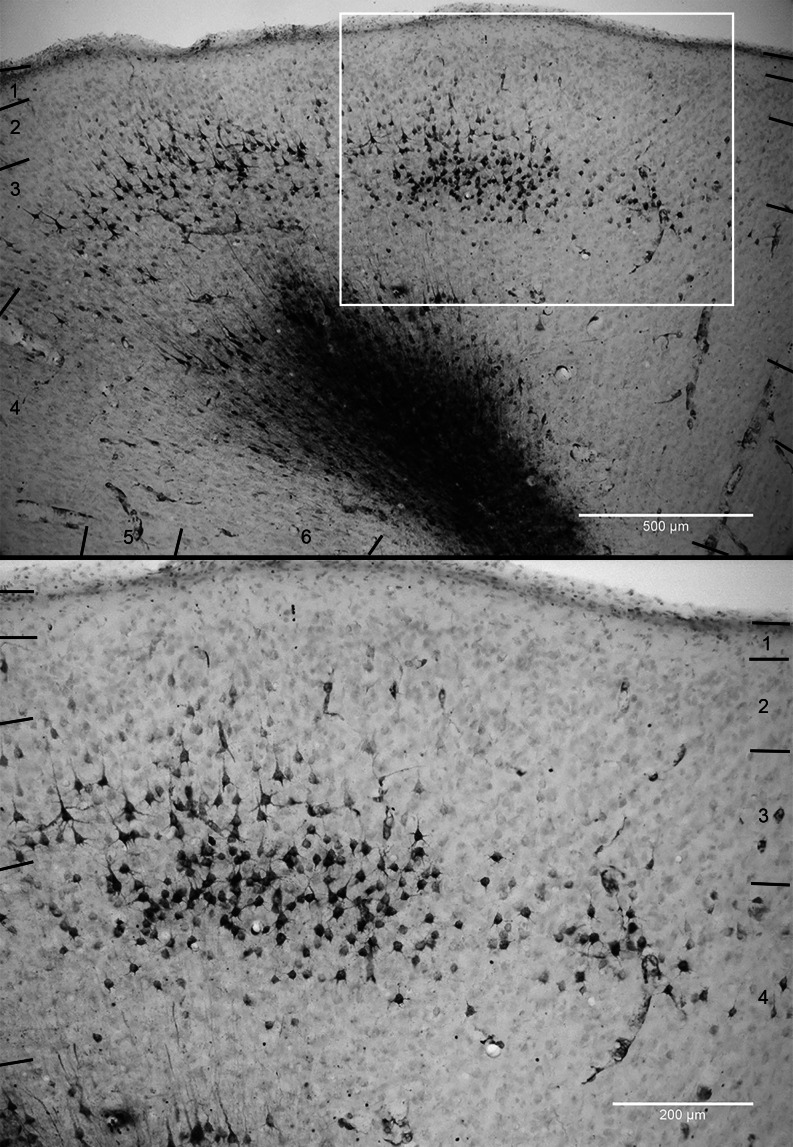
Patchy distribution of retrogradely labeled layer 3/4 cells in area 17. *Top*: Coronal section through the medial bank of the lateral gyrus (bending towards the top of the gyrus on the left) with Nissl stain. Retrogradely labeled neuronal cell bodies and some blood vessel outlines appear black. Large dark stain at injection site in layer 6. Numbers: cortical layers. Scale bar 500 μm. *Bottom*: Zoom-in of the white frame in the top image: A cluster of retrogradely labeled neurons at the layer 3/4 border. Neuronal cell bodies and parts of their most proximal dendrites are visible. Scale bar 200 μm

This distribution of labeled neurons across the cortical layers was unexpected because neurons in the supragranular layers made the smallest contribution to the asymmetric synapses in layer 6 of all layers in the Binzegger diagram (apart from layer 1, in which no spiny neuron had been reconstructed). The retrogradely labeled neurons we identified in layers 2 and 3 thus provide an additional source of input to layer 6. Interestingly, the neurons labeled retrogradely from layer 6 were not distributed evenly across layers and sublamina. In the ‘local column’ directly above the injection site, all layers except layer 1 contained labeled neurons and the labeled neurons were found throughout the whole depth of layer 3. The largest fraction of the labeled neurons lateral to the ‘local column’ was restricted to the layer 3/4 border region and in some cases were grouped in clusters (see Fig. 6. These lateral clusters are also evident in the series of coronal sections of Fig. 2a: They were 400 to 800 μm wide (horizontal spread in coronal sections), 150 to 250 μm thick (the most superficial neurons at a depth of 300 to 400 μm from the brain surface) and their centers were 400 to 800 μm apart. (Cluster dimensions were not measured systematically in all cats, this is a qualitative description of their scale based on the most distinct clusters in cat 1).

### Candidate neurons

**Fig. 7:**
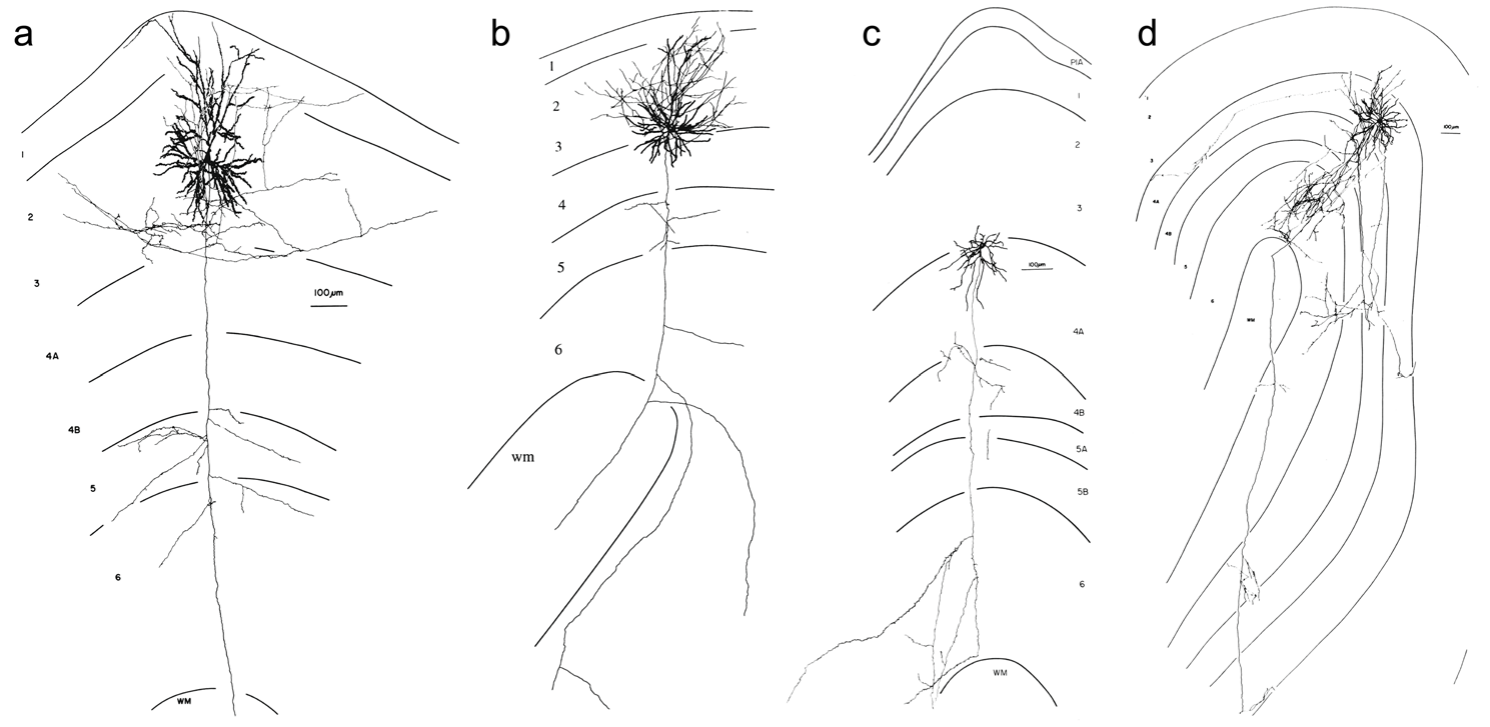
Candidate layer 3/4 neurons projecting to layer 6. (**a**) Canonical layer 3 pyramidal cell with axon branches in layers 3 and 5. (**b**) Layer 3 pyramidal cell at the layer 3/4 border with additional axon branches running for more than 1 mm in layer 6. (**c**) Layer 4 spiny stellate cell with many axon branches in layer 6. (**d**) Layer 4 spiny stellate cell with a large axon cluster in layer 6 and long horizontal branches in layers 4, 5 and 6

The layer 3/4 border cells that innervate layer 6 are different from the canonical superficial layer pyramidal cell whose descending axon forms a collateral arbor in layer 5 before entering the white matter (Fig. 7a) (Gilbert 1983; Martin and Whitteridge 1984; Martin et al. 2017). Some of these have additional collaterals in layer 6 (see Fig. 7b). The canonical spiny stellate cells have axon arbors either restricted to layer 4 or arborizing in layers 2 and 3. The canonical star pyramid of layer 4 sends occasional axons to the deep layers, but most of its axon forms clusters in layers 3 and 4 (Martin and Whitteridge 1984; Martin et al. 2017).

In search of further evidence for direct projections from the layer 3/4 border to layer 6, we examined our unpublished in-house database of neurons from cat area 17 that had been physiologically-characterized in vivo and intracellularly-filled with HRP. Of the 22 spiny neurons in layers 3 and 4 A with axonal arbors in deep layers, 17 targeted layer 5 but not layer 6 (although 11 of these had an axon entering the white matter) and 5 had axon branches in layer 6. Three of these five neurons with axon branches in layer 6 had longer axon collaterals in layer 6 than in layer 5; they consisted of a pyramidal cell in deep layer 3 and two layer 4 spiny stellate cells (see Fig. 7b, c, d).

The layer 3 pyramidal cell of Fig. 7b was just at the layer 3/4 border and its axon collateral traveled for more than a millimeter horizontally in layer 6. The spiny stellate cell in upper layer 4 A (Fig. 7c) was relatively sparsely-filled, but had several axon branches in layer 6 continuing for several 100 μm. The other spiny stellate cell (Fig. 7d), in contrast, was well-filled. It had three axon clusters in layer 6: a large and dense one below the soma, a smaller one from a branch in layer 4 descending at a distance of around 1 mm from the soma to the deep layers and another small one from a horizontal branch passing through the white matter and layer 6. This axon continued further up again to make another small arbor in layer 5 and ended in a small arbor in upper layer 4 at a horizontal distance of 3 mm to the soma.

The three reconstructed neurons in Fig. 7b, c, d are likely candidates for the ‘layer 3/4 to layer 6’ neuron types, since their axon distribution would have allowed them to take up HRP from an injection site in layer 6. Notably, the laminar preference of their axonal clusters was very different from the five canonical ‘layer 3 to layer 5’ pyramidal cells used by Binzegger et al. for their calculations.

### Estimating relative size of ‘layer 3/4 to layer 6’ cell population

Binzegger et al’s (2004) calculations were based on the contribution of neurons in area 17 and the X- and Y-type thalamocortical axons. Synapses arising from all other external sources were not included, but of course these necessarily make some contribution to the ‘dark matter’. From our calculations of the FLN, we found that about 80% of the neurons that project to layer 6 were in area 17, most of which were in layers 3 and 4, with a smaller component from other layers within the local column. We thus examined the effect of integrating two additional spiny cell types into the Binzegger diagram: a ‘layer 3 to layer 6’ pyramidal cell, p3(L6), and a ‘layer 4 to layer 6’ spiny stellate or pyramid, sp4(L6). Based on the FLN of area 17 in our retrograde tracing experiments, the proportion of asymmetric synapses in layer 6 that was accounted for by the local circuit was set to 81.1 ± 14.2% (the remaining 18.9% coming from other visual areas). Since the Binzegger diagram only accounted for 30% of these synapses, our estimate would require nearly a tripling of the local input to layer 6.

If the neurons around the layer 3/4 provide these additional asymmetric synapses in layer 6, how many synapses would that be? In the Binzegger diagram, each p2/3 cell (the canonical superficial layer pyramidal cell) formed 78 synapses in layer 6. With 8.25 · 10^6^ spiny neurons in layer 2/3 of area 17, this corresponds to a total of 6.41 · 108 synapses in layer 6. Each of the three spiny layer 4 cells of the Binzegger diagram, ss4(L2/3), ss4(L4) and p4, made on average 85 ± 25 synapses in layer 6. And when multiplied by 8.74 · 10^6^, the total number of spiny neurons in layer 4, the spiny neurons of layer 4 formed in total 7.4 · 10^6^ synapses in layer 6 (calculated from supplementary Table 5 in Binzegger et al. (2004). In order to reach a percentage of unassigned synapses below 20%, 1.1 · 10^10^ additional synapses are required.

We have to estimate the proportions of spiny neurons that p3(L6) and sp4(L6) could have in their respective layers. Because we now have four types of spiny neurons in layer 4, but lack further information on their relative proportions (Binzegger et al. 2004), the simplest assumption is that the sp4(L6) cells constitute a quarter of the population in layer 4. The proportion of p3(L6) cells is likely to be less than half of all pyramidal neurons in layers 2/3, since the superficial layer pyramidal cells with collateral arbors in layer 5 seem to dominate in our intracellular studies (Martin and Whitteridge 1984; Martin et al. 2017). Away from the local column, the retrogradely-labeled neurons clustered only in the lower part of layer 3.

**Fig. 8:**
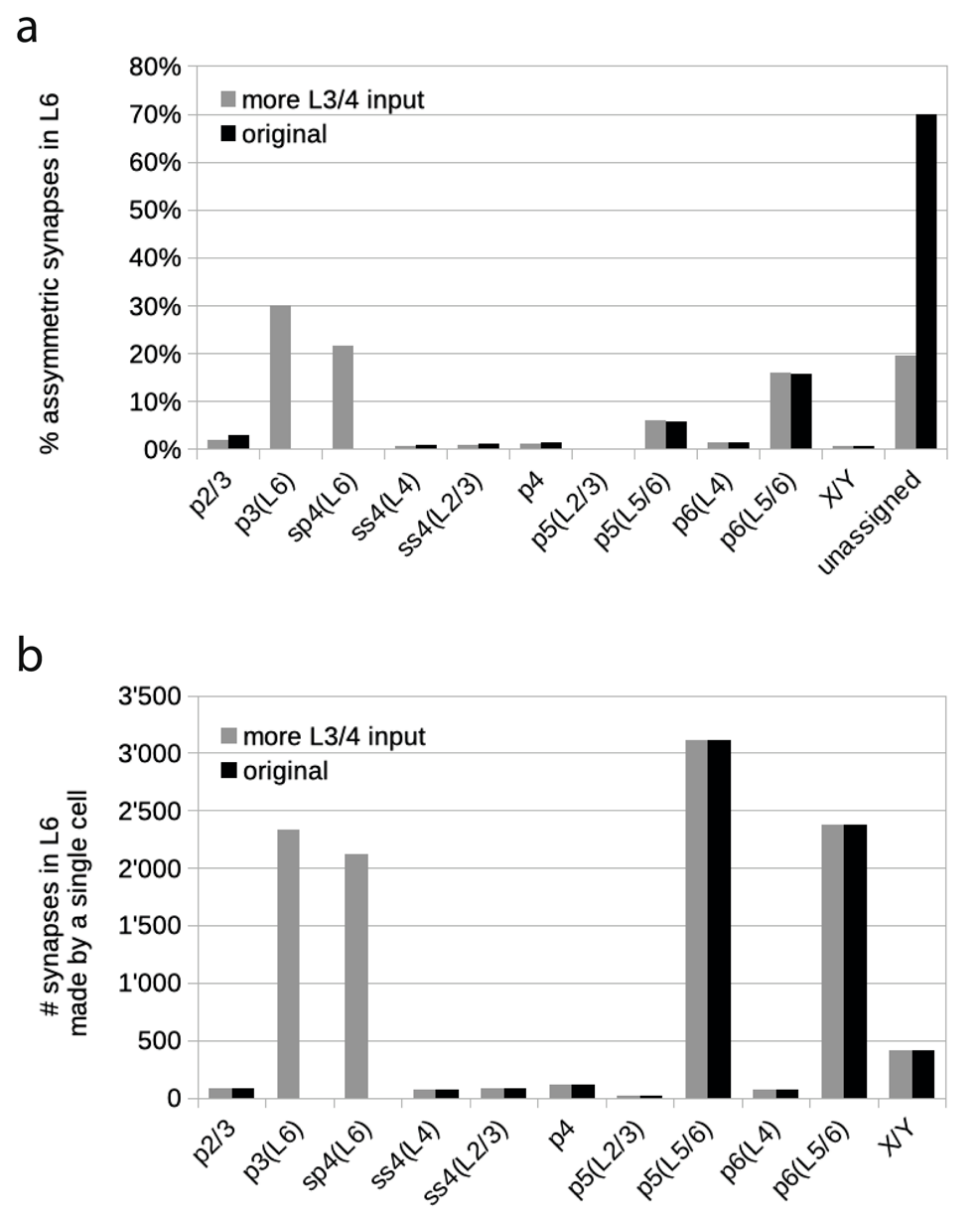
Adding the layer 3/4-to-layer 6 input into the Binzegger diagram. (**a**) Comparison of the percentage of total asymmetric synapses in layer 6 of the Binzegger diagram (labeled ‘original’) with the hypothetical case where the inputs come from the new cell types identified in the present study. The layer 3 pyramids p3(L6) are assumed to provide 30.0% and the layer 4 type sp4(L6) 21.7% of asymmetric synapses in layer 6. These new inputs would leave only 19.2% unassigned synapses (based on our FLN of 81% for area 17), compared to 70% unassigned in the Binzegger diagram. (**b**) Total number of synapses that a single cell of each given cell type makes in layer 6. Each ‘standard’ excitatory cell of layers 3 and 4 in the Binzegger diagram (labeled ‘original’) made on average 78 (p2/3) and 85 (mean of ss4(L2/3), ss4(L4) and p4) synapses in layer 6. In the hypothetical case given in (**a**), p3(L6) cells would each form 2329 synapses in layer 6 and sp4(L6) cells would each form 2115 synapses in layer 6

Figure 8 shows a hypothetical scenario that would result in only 19.2% of unassigned asymmetric synapses in layer 6 versus the 70% in the estimates of Binzegger et al. (2004). Based on our FLN of 81% for area 17, the 19.2% unassigned synapses would thus originate from neurons outside area 17. In this hypothetical case, p3(L6) cells would make up one third of spiny layer 2/3 neurons and sp4(L6) cells 25% of spiny layer 4 neurons. Each of these cells (p3(L6) and sp4(L6)) would have to provide 25–30 times as many synapses in layer 6 than the ‘standard’ spiny neurons of the Binzegger diagram in their respective layers. In numbers, this means that p3(L6) cells would have each to make 2329 synapses and sp4(L6) cells would each have to make 2115 synapses in layer 6. Such large numbers of synapses are not inconceivable, e.g. Binzegger et al. (2004) found that each p5(L5/6) type of layer 5 cell forms an average of 3109 synapses in layer 6 and the p6(L5/6) type of layer 6 pyramidal cell forms 2378 synapses in layer 6.

**Fig. 9:**
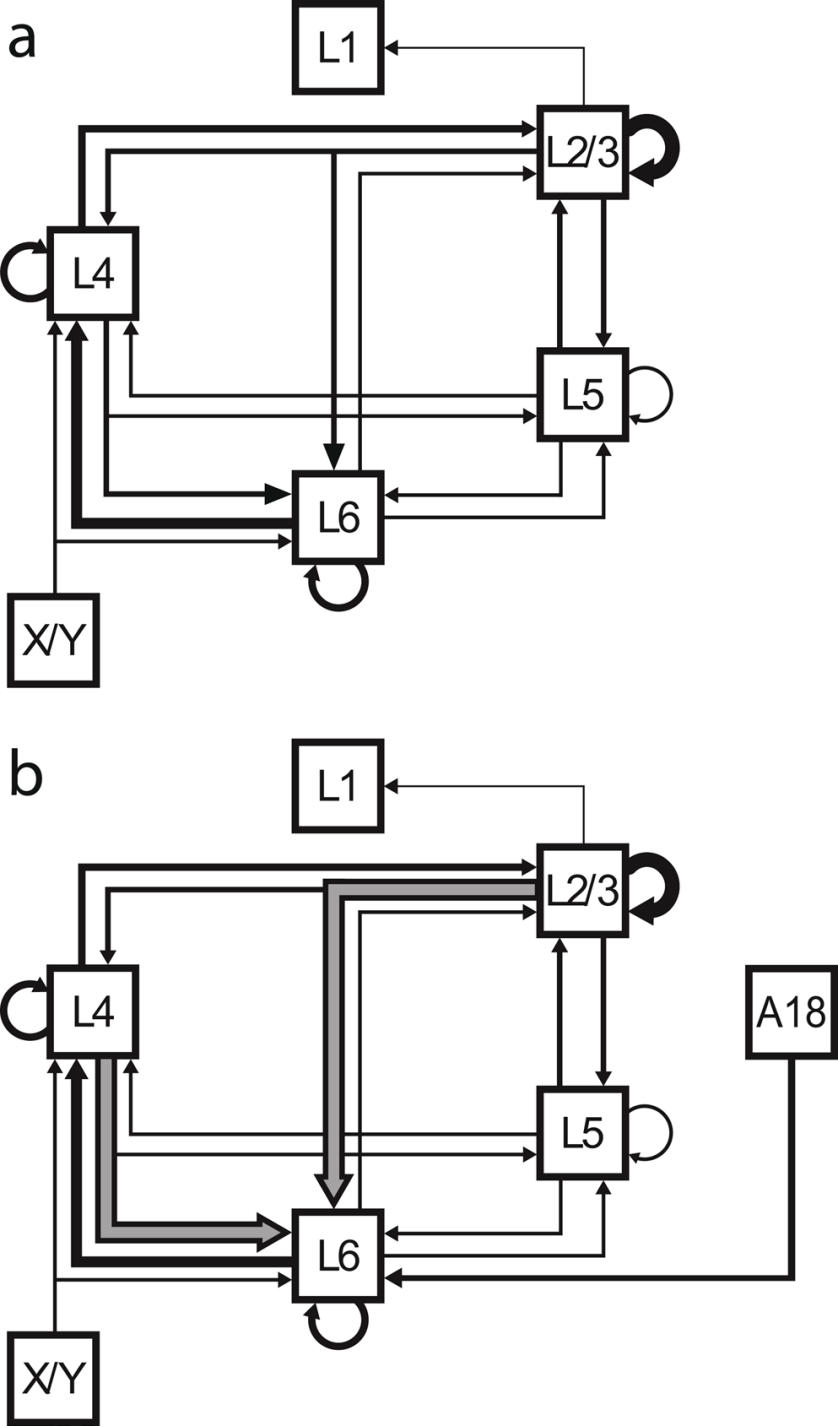
Schematic of the excitatory interlaminar connections of cat V1. (**a**) Modified from the original diagram. Thicker arrows indicate higher weight connections. (**b**) Updated diagram: Gray arrows with black outlines mark connections that are stronger according to our tracing experiments than previously estimated in the Binzegger diagram. L1–6: Layers 1–6. X/Y: thalamic input from the lateral geniculate nucleus (LGN). A18: area 18. adapted from Binzegger et al. (2004), Fig. 12A

## Discussion

Our retrograde tracer injections in layer 6 of cat primary visual cortex revealed that distant brain areas contribute a small fraction of the input to layer 6. Instead we found that more than 80% of all labeled neurons were located in the vicinity of the injection sites. The most striking feature of this local input was that the source neurons were around the layer 3/4 border. This population has not been detected before, despite decades of work on the circuits of cat’s visual cortex. Thus, the local excitatory circuit diagram (see Binzegger et al. 2004; Fig. 12A) needs to be updated with more numerous connections from layers 3 and 4 to layer 6 (Fig. 9).

### Injection sites and tracer

The (post-)lateral gyrus was the ideal place to target layer 6 of area 17, because the deepest cortical layer thickens as it wraps around the gyrus and is also easily accessible from the brain surface. Nevertheless, placing tracer injections in single cortical layers is a delicate matter. The pipette tip needs to be exactly positioned and the injected volume carefully balanced. If the injection is too small, not many neurons will be labeled, and if it is too large, the tracer may spread to other layers. While we cannot exclude some diffusion of HRP to layer 5 or the white matter, the pattern of labeled neurons in area 17 was quite repeatable and not what would be expected if there were uptake from layer 5 or white matter fibers. Also the pattern of labeled neurons in the LGN laminae agree with known projections from LGN to layer 6: The LGN Y-axons have a collateral projection to layer 6 (LeVay and Ferster 1977; Ferster and Levay 1978; Humphrey et al. 1985; Freund et al. 1985). The C laminae also contain Y-type cells that project directly to area 18 and some branch to innervate both area 17 and 18 (Humphrey et al. 1985; Freund et al. 1985). Our finding that injections at the area 17/18 border led to the highest proportion of labeled neurons in C versus A laminae are in line with this projection.

### Ipsilateral input from long-range connections

A principle of neocortical wiring is that there are abundant local connections and sparse long-range connections (see e.g. Batardiere et al. 1998; Barone et al. 2000; Schüz et al. 2006; Stepanyants et al. 2009; Markov et al. 2011, 2013a, b; Markov and Kennedy 2013; Anderson and Martin 2016; Horvát et al. 2016). From the perspective of wiring cost, if the vast majority of a neuron’s postsynaptic partners are in its immediate vicinity, long-distance connections and hence brain volume are significantly reduced and signal transmission distances are optimized.

The superficial cortical layers are typically identified as the source of ‘feedforward’ connections, while conversely, the deep layers are the source of feedback connections. The quantitative ‘distance rule’ developed for the macaque cortex states that the ratio of deep vs. superficial labeled neurons is a function of the relative positions of the source and recipient areas in the cortical hierarchy. Adjacent areas have similar numbers of labeled neurons in superficial and deep layers, but as the hierarchical distance increase, the difference in the numbers in the superficial vs. deep layers becomes more and more divergent (Markov and Kennedy 2013). This ‘distance rule’ is not apparent in the projection to layer 6 from adjacent area 18, where the majority of labeled neurons were located in the superficial layers, which the distance rule would classify as a feedforward projection from much lower in the hierarchy. By contrast, in area 19, the majority of labeled neurons were in the deep layers, which the distance rule would classify as a feedback projection from an area much higher in the hierarchy (see Fig. 5).

### Contralateral input from the transition zone is retinotopic

The callosal fibers between the primary visual cortices of the two hemispheres have been found to interconnect preferentially neurons with receptive fields lying along the representation of the vertical meridian, which demarcates the boundary between areas 17 and 18. The strongest callosal projections were reported to originate in layer 3 pyramidal cells (Choudhury et al. 1965; Hubel and Wiesel 1967; Harvey 1980; Squatrito et al. 1981; Voigt et al. 1988; Buhl and Singer 1989; Payne 1991; Payne and Siwek 1991; Houzel et al. 1994, 2002; Houzel and Milleret 1999). In agreement with these previous studies, the majority of our labeled neurons in the contralateral visual cortex were located in layer 3 of areas 17 and 18.

The horizontal spread of contralaterally labeled neurons we observed matched the spread of retrogradely labeled neurons reported by Payne (1991; see Fig. 3). Only the density of labeled neurons was lower in our case, since we had injected layer 6 and not layers 3 and 4, which receive the densest transcallosal projection (Houzel et al. 1994; Rochefort et al. 2009). The differences we observed after injections into area 17 or the area 17/18 border fit the connectivity rule described by Olavarria (1996): The area 17/18 border region (‘transition zone’), which represents the central part of the ipsilateral visual field (Hubel and Wiesel 1962; Whitteridge and Clarke 1982; Payne 1990), receives input from the region in the contralateral hemisphere where the same part of the visual field is represented, namely from the regions in areas 17 and 18 surrounding the transition zone. According to the same retinotopy rule, injections clearly inside area 17 (on top of the lateral gyrus) labeled neurons in the contralateral transition zone (Olavarria 1996; Stryker and Antonini 2001).

## Distribution of labeled neurons within area 17

It was not previously appreciated that the input to a point in layer 6 in area 17 arises not only from neurons within the same local column, but also from neurons at distant sites whose receptive fields would be at least 10 degrees away from those of neurons at the injection site (Kalia and Whitteridge 1973; Gilbert 1977; Tusa et al. 1978; Bullier et al. 1984). Layer 6 does contain some neurons with the largest receptive fields recorded in area 17 (10–20 deg. in length; Gilbert 1977; Grieve and Sillito 1995*)*.

One hypothesis (Gilbert 1983; Gilbert and Wiesel 1983a) for the large fields in layer 6 is that they are formed from the convergent input from the complex cells in layer 5B, whose extensive axons innervate layer 6 (Gilbert and Wiesel 1979; Martin and Whitteridge 1984). Binzegger et al. (2004) calculated that this input from layer 5 is modest, which is consistent with the results of a physiological study in rat visual cortical slices using glutamate uncaging (Zarrinpar and Callaway 2006). Of course, in vivo all the inputs are intact, so many more distant neurons contribute to the receptive field properties of a given layer 6 neuron. Here our discovery that a point in layer 6 receives input from neurons of distant retinotopic locations within area 17, identifies another input that likely contributes to the formation of large receptive fields in some layer 6 neurons.

### Candidate neurons projecting from upper layers to layer 6

Apart from our own reconstructions of layer 3/4 neurons reported here, at least two other published examples of a neuron would fit the proposed ‘layer 3/4 to layer 6’ pattern. Stepanyants et al. (2008) reconstructed a layer 3 pyramidal cell at the layer 3/4 border of cat area 17 whose axonal arbors in the deep layers are not restricted to layer 5, but descend through layer 6 and cross the white matter to form multiple clusters on the lateral bank of the lateral sulcus close to the area 17/18 border, both in deep and superficial layers (see Fig. 2 in Stepanyants et al. 2008). Hirsch et al. (1995) also described a spiny stellate cell with horizontal axon ramifications in layer 6.

These examples seem to be the exception in the published pyramidal cell pool, however. As in the data used for the Binzegger diagram, the layer 2 and 3 pyramidal cells reconstructed earlier by Martin and Whitteridge (1984*)* all showed the typical standard pattern of selective axon arbors in layers 3 and 5; and four out of six neurons had an axon descending straight into the white matter. Similarly, the sample of reconstructed cortical cells used by Gilbert to propose an interlaminar microcircuit of excitatory connections in cat primary visual cortex contained just the classical layer 3 to layer 5 pyramidal cell in layer 3 (Gilbert and Wiesel 1979, 1983a, b; Gilbert 1983).

Other reconstructed layer 3 pyramidal neurons in cat cortex (cited in http://neuromorpho.org/) all matched the canonical form used by Binzegger et al. (2004): in the deep layers, their axons arborized mainly in layer 5 and may have continued through layer 6 with a non-branching axon to reach the white matter (Hirsch et al. 2002; Martinez et al. 2005; Volgushev et al. 2006; Budd et al. 2010). (Note that the neuron reconstructed by Stepanyants et al. (2008) is actually listed in the neuromorpho database, but linked to a different publication: http://neuromorpho.org/neuron_info.jsp?neuron_name=980804axden.)

Probably the most extensive selection of layer 3 pyramidal cells in cat V1 was reconstructed for the analysis of cortical ‘daisies’ – the axon clusters of pyramidal cells in layer 2, 3, and 4 (Martin et al. 2017). Most of these neurons had the canonical layer specificity of superficial layer pyramidal cells: Of the 45 layer 3 pyramidal cells and the five ‘star’ pyramidal cells at the upper border of of layer 4, only four neurons had more than 5% of their boutons in layer 6. It may be that the sublaminar and patchy distribution of these neurons has resulted in them being under-sampled, yet the few instances mentioned above show that there does exist a population of layer 3/4 cells with more extensive axonal arbors in layer 6 than the spiny neurons used for the original Binzegger diagram.

### How to incorporate these neurons into the Binzegger diagram?

In order to increase the weight of the excitatory connection from layers 3 and 4 to layer 6 in area 17, we calculated the average number of synapses a spiny cell in layer 3 and 4 would have to form in layer 6. If the ‘layer 3/4 to layer 6’ neurons only constitute a small fraction of layer 3/4 neurons, each of the ‘layer 3/4 to layer 6’ neurons is required to form 40 times more synapses in layer 6 than the original layer 3/4 cells. While this seems a large factor, there are other examples of similar differential laminar preferences between different neuron types of the same layer in the cortex. For example, the average p6(L5/6) neuron forms 33 times more synapses in layer 6 than the average p6(L4) neuron, while the average p6(L4) even forms 58 times more synapses in layer 4 than p6(L5/6). Furthermore, we do have evidence from neuron reconstructions that neurons in layers 3 and 4 with dense axon arbors in layer 6 exist.

Clearly, adding these ‘new’ neuron types in layers 3 and 4 that contribute more synapses to layer 6 will change the proportions of the original spiny cell types in layers 3 and 4. This in turn will affect the number of synapses in other layers, unless the only difference between the original and the added neuron types in layers 3 and 4 is in the number of synapses they form in layer 6.

In layers 2 and 3, the Binzegger diagram actually accounted for slightly more than 100% of asymmetric synapses, of which 71% came from layer 2/3 pyramidal cells. On the other hand, 32% of asymmetric synapses in layer 4 remained unassigned. We have since shown that the layer 3/4 border cells provide more synapses to layer 4 that those higher in the superficial layers (Martin et al. 2017). Taking this observation into account partly resolves this imbalance of a small excess of asymmetric synapses in layer 3 and a deficit in layer 4.

#### Functional considerations and further refinements

Lower layer 3 and upper layer 4 receive direct thalamic input from the LGN, predominantly from Y cells (Bullier and Henry 1979; Martin and Whitteridge 1984). The neurons at the layer 3/4 border could thus forward this information on fast stimuli directly to layer 6, as a short-cut connection bypassing the classical feedforward path via layer 3 and layer 5, as a parallel ‘inner loop’ of the interlaminar cortical circuit. This is not dissimilar to the circuit in macaque area 17, where the magnocellular input to 4Calpha is relayed to layer 4B and thence to output layers 5 and 6, without first being relayed through the superficial cortical layers (Bullier and Henry 1980; Anderson et al. 1993). Layer 4B of Brodmann (1905*)* is not a thalamorecipient layer in the monkey and indeed, in the later nomenclature of Hassler (1966*)* was called layer IIIc. In monkey V1, layer 6 pyramidal cells receive excitatory inputs from specific sublaminae of layers 3 and 4 (Anderson et al. 1993; Briggs and Callaway 2001), whereas in rat V1 layer 6 receives small inputs from the layers above (Zarrinpar and Callaway 2006).

This interpretation of the circuit predicts that the ‘layer 3/4 to layer 6’ subpopulation preferentially targets specific neuron types in layer 6 with a preference for stimuli of high temporal and low spatial frequency typical of the magnocellular or Y-pathway, such that the different pathways from superficial to deep layers would be kept largely separate (Bullier and Henry 1979, 1980; Martin and Whitteridge 1984). The recurrent excitatory pathway from layer 6 also segregates into at least three pathways (Katz 1987). Hirsch et al. (1998) found that the axons of simple layer 6 pyramidal cells mainly targeted layer 4 (where simple cells predominate), whereas the axons of complex layer 6 pyramidal cells targeted layers 2/3 and 5 (where complex cells predominate). A third pathway is formed by the claustral-projecting cells, which project within layer 6 itself.

Peters’ rule is useful for assessing average connectivity and its associated variance and is essential for assessing the existence of White’s exceptions. For example, da Costa and Martin (2009*)* later identified another ‘White’s exception’ in their study of the thalamic input to layer 6 corticothalamic cells, which formed synapses with the thalamic afferents mainly on their basal dendrites and only rarely on their apical dendrites in the major thalamo-recipient zone of layer 4. Further, in their correlated LM-EM study of layer 3 pyramidal cell axons in layer 3, Koestinger et al. (2017a) were able to show that the variance in spiny vs. smooth targets was the same magnitude as that encountered in a simulated quasi-random walk through the neuropil. Koestinger et al.’s (2017b*)* correlated LM-EM study of the translaminar projection to layer 5 of the same layer 3 pyramidal cells showed how diverse the synaptic targets were at single collateral resolution and emphasized how difficult it is to identify circuits at sublaminar resolution, particularly when the apical dendrites of deeper pyramidal cells pass through the layer of axonal arborisation.

The direct projections to layer 6 we have discovered add yet more to this circuit complexity. While this new pathway is consistent with the recurrent architecture of the ‘canonical circuit’ proposed by Douglas et al. (1989), refining the quantitative description pioneered by Binzegger et al. (2004) and unpicking the synaptic specificity of these descending pathways by taking into account cell-type specificities and sublaminar preferences in connectivity clearly remains a formidable challenge.

### Electronic supplementary material

Below is the link to the electronic supplementary material.

Supplementary Material 1

## Data Availability

The datasets generated during and/or analysed during the current study are available from the corresponding author on reasonable request.
